# Abnormal brain spontaneous activity in major depressive disorder adolescents with non-suicidal self injury and its changes after sertraline therapy

**DOI:** 10.3389/fpsyt.2023.1177227

**Published:** 2023-06-13

**Authors:** Linqi Dai, Xiaoliu Zhang, Renqiang Yu, Xingyu Wang, Fei Deng, Xue Li, Li Kuang

**Affiliations:** ^1^Department of Psychiatry, The First Affiliated Hospital of Chongqing Medical University, Chongqing, China; ^2^Department of Radiology, The First Affiliated Hospital of Chongqing Medical University, Chongqing, China

**Keywords:** superior frontal, superior occipital, orbital middle frontal, lingual, mALFF, sertraline therapy, non-suicidal self-injury, adolescent depression

## Abstract

**Background:**

Non-suicidal self-injury (NSSI) commonly occurs among adolescents with major depressive disorder (MDD), causing adverse effects on the physical and mental health of the patients. However, the underlying neurobiological mechanism of NSSI in adolescents with MDD (nsMDDs) remains unclear, and there are still challenges in the treatment. Studies have suggested that sertraline administration could be an effective way for treatment.

**Methods:**

To verify the effectiveness and to explore the neurobiological processes, we treated a group of adolescents with nsMDDs with sertraline in this study. The brain spontaneous activity alteration was then investigated in fifteen unmedicated first-episode adolescent nsMDDs versus twenty-two healthy controls through the resting-state functional magnetic resonance imaging. Besides the baseline scanning for all participants, the nsMDDs group was scanned again after eight weeks of sertraline therapy to examine the changes after treatment.

**Results:**

At pre-treatment, whole brain analysis of mean amplitude of low-frequency fluctuation (mALFF) was performed to examine the neuronal spontaneous activity alteration, and increased mALFF was found in the superior occipital extending to lingual gyrus in adolescent nsMDDs compared with controls. Meanwhile, decreased mALFF was found in the medial superior frontal in adolescent nsMDDs compared with controls. Compared with the pre-treatment, the nsMDDs group was found to have a trend of, respectively, decreased and increased functional neuronal activity at the two brain areas after treatment through the region of interest analysis. Further, whole brain comparison of mALFF at pre-treatment and post-treatment showed significantly decreased spontaneous activity in the orbital middle frontal and lingual gyrus in adolescent nsMDDs after treatment. Also, depression severity was significantly decreased after treatment.

**Conclusion:**

The abnormal functional neuronal activity found at frontal and occipital cortex implied cognitive and affective disturbances in adolescent nsMDDs. The trend of upregulation of frontal neuronal activity and downregulation of occipital neuronal activity after sertraline treatment indicated that the therapy could be effective in regulating the abnormality. Notably, the significantly decreased neuronal activity in the decision related orbital middle frontal and anxiety-depression related lingual gyrus could be suggestive of reduced NSSI in adolescent MDD after therapy.

## 1. Introduction

Major depressive disorder (MDD) is a common psychiatric disorder that is characterized by persistent low mood and loss of interest or pleasure in all activities, and these symptoms could finally lead to clinical sufferings (1). As a transitional stage from childhood to adulthood, the period of adolescence has a high prevalence of the psychiatric disorders such as MDD. And the adolescent depression could also increase the risk of depression later in the adulthood (2). Additionally, MDD has a significant association with self-injury and suicide (3). Non-Suicidal Self-Injury (NSSI) is defined as deliberately damaging one’s own body without intention to die (4). According to the DSM-V, subjects with NSSI would be engaged in self-injury behavior five or more days within the past year to gain relief from the negative feelings or cognitive states (5). It has been further indicated that NSSI should be considered as a specific predictor of suicide (6). With the high incidence rate and suicide risk, NSSI has been attracted widespread attention among scholars. However, the underlying neurobiological mechanism is still unclear, and there are still challenges in the treatment for this group of population.

Resting-state functional magnetic resonance imaging (rs-fMRI) is a non-invasive neuroimaging technique which is based on the spontaneous fluctuations of the blood oxygen level dependent (BOLD) signal. It has been widely used in psychiatry disorder research as it could help uncover the neuronal activity pattern. The amplitude of low frequency fluctuation (ALFF) is one of the measurements commonly used to reflect the intensity of brain regional spontaneous activity (7). Moreover, ALFF could reflect the cyclic modulation of gross cortical excitability and long distance neuronal synchronization (8, 9). Previous studies on adolescent MDD have found impaired functional activation associated with emotional processing and regulation (10). Also, the deficits in developing neural systems such as visuo-spatial attention and sustained visual attention in adolescent MDD patients were also suggested (11, 12). While, there were few studies on adolescent MDD with NSSI.

In a functional study of NSSI in female adolescents without MDD, Plener et al. reported that NSSI patients showed altered neural activity pattern at limbic and fronto-occipital areas when watching the emotional and NSSI pictures (13). Our previous study also found that MDD adolescents with NSSI showed significantly increased ALFF signals in the frontolimbic brain regions compared with those patients without NSSI (14, 15).

Untill now, there is no recommended plan for NSSI treatment, and the optimal treatment for NSSI in adolescent MDD is still being explored. Several interventions appear to have an effect of reducing NSSI, including psychotherapy such as dialectical behavior therapy and emotion regulation group therapy, antipsychotics, and selective serotonin reuptake inhibitors (SSRIs). There remains a paucity of well-controlled studies investigating treatment efficacy of NSSI (16). Of the functional neuroimaging studies in NSSI, Santamarina-Perez et al. (17) Explored 4 weeks of psychotherapy among adolescents with NSSI, and they found that the strength of the amygdala-prefrontal connectivity could predict the efficacy of treatment. Cullen et al. (18) explored the circuit-level changes following an eight-week trial of N-acetylcysteine in female adolescents with NSSI, and they suggested that amygdala and nucleus accumbens-based circuits could serve as potential targets for treatment. These studies have demonstrated that NSSI in adolescents have neural circuits changes linked with the efficacy of treatment. However, these participants were not diagnosed with MDD.

Second-generation antidepressants are widely used for the treatment of MDD, including SSRIs, are recommended as the first-line medication for depression (19). Sertraline is an SSRI that has been used for the depression treatment. Studies have demonstrated that sertraline treatment for children and adolescents’ MDD is effective and well-tolerated (20, 21). In the present study, we used the sertraline treatment for adolescent MDD with NSSI (nsMDDs). The rs-fMRI data was acquired at baseline and after treatment to explore the neurobiological mechanism and processes in adolescent nsMDDs. The mean ALFF (mALFF) analysis of imaging data was applied to improve the signal detection. Here, we hypothesized that: (i) The adolescent nsMDDs would have mALFF alterations at brain areas involved with cognitive and affective processing and (ii) The sertraline treatment would regulate the abnormal functional neuronal activity at the altered brain regions through the inhibition of selective serotonin reuptake in adolescent nsMDDs.

## 2. Materials and methods

### 2.1. Participants

Fifteen adolescents with nsMDDs were recruited from the outpatient clinic of the Department of Psychiatry at the First Affiliated Hospital of Chongqing Medical University, China, from January 2021 to March 2022. This study was approved by the Human Research and Ethics Committee of the First Affiliated Hospital of Chongqing Medical University (no. 2021-546). Written informed consent was obtained from all adolescents and their caregivers.

All the patients were evaluated by two experienced psychiatrists using the Mini International Neuropsychiatric Interview for Children and Adolescents (MINI-kid). The diagnostic criteria for NSSI were based on the Diagnostic and Statistical Manual of Mental Disorders, the fifth edition (DSM-V). These patients did not take any psychotropic drugs before participating in this study. The primary inclusion criteria were as follows: (i) Han nationality, aged 12–17 years, and right-handed, (ii) first-episode depression with no history of antidepressant treatment or psychotherapy, (iii) reported NSSI events five times or more in the past 12 months and at least once NSSI in the last month according to the Ottawa self-injury inventory (OSI), (iv) exhibited a Hamilton Depression Scale (HAMD-17) score > 17, (v) no MRI scanning contraindication such as metal implants, (vi) no history of brain trauma or other serious physical illnesses, (vii) no substance abuse or dependence, and (viii) no history of any other mental health disorders. The severity of depression was assessed using the 17-item Hamilton Depression Scale (HAMD) (all patients’ score > 17). The patients were once assessed at baseline and then reassessed after 8 weeks of antidepressant medication treatment.

In addition, twenty-two healthy controls (HC) matched for age, gender and education level were recruited from the local school. The inclusion criteria were Han nationality, aged 12–17 years, right-handed, HAMD score < 7, and absence of any severe mental or physical illness.

### 2.2. Antidepressant medication protocol

Sertraline was selected as the main antidepressant drug with an initial dose of 50 mg per day, and the maximum dose was 100–200 mg per day depending on the patients’ condition. The medication continued for 8 weeks.

### 2.3. Imaging protocol

All MR images were acquired with 3.0-T GE Signa HDxt (General Elecric Healthcare, Chicago, Illinois, United States) scanner equipped with a standard 8-channel head coil. Contiguous sagittal T1-weighted images across the entire brain were acquired with a fast gradient echo (FGRE) sequence: time of echo (TE) = 3.1 msc, time of repetition (TR) = 8 msc, flip angle = 12°, field of view (FOV) = 240 mm, voxel size = 0.938 × 0.938 × 1 mm^3^, no gap. The rs-fMRI data were obtained using an echo-planar image (EPI) pulse sequence with the following parameters: 33 axial slices, TE = 40 msc, TR = 2000 msc, in-plane resolution = 64 × 64 pixels, flip angle = 90°, FOV = 240 mm, voxel size = 3.75 × 3.75 × 4 mm^3^, no gap. A total of 240 time points was obtained over 8 min. All participants including both adolescent nsMDDs and HC underwent baseline MR scanning. After 8 weeks of treatment, the adolescent nsMDDs was scanned once again.

### 2.4. Imaging processing and analysis

Two experienced imaging professionals performed quality control of the original data, and no excessive head movements or abnormal signals were observed in any image. Then, imaging processing was performed with SPM12^1^ and RESTplus toolkits^2^ within MATLAB 2013b (MathWorks, Natick, MA, United States).

The procedures were as follows: (i) the original structural and functional image data were firstly converted into NII and NIFTI formats, (ii) the first 10 time points were removed to eliminate nonequilibrium effects of magnetization, (iii) slice timing and head motion correction were then performed to exclude the participants whose head movements exceeded 3 mm translation or 3 degree rotation in any direction, (iv) functional images were spatially normalized to the Montreal Neurological Institute (MNI) EPI template and resampled as 3 × 3 × 3 mm^3^, (v) a standardization smoothing through the Gaussian filter of 6-mm full width at half maximum (FWHM) was performed to reduce the influence of spatial noise and the differences of brain structure among subjects, and (vi) nuisance regression was performed using the six head motion parameters, white matter, and cerebral spinal fluid BOLD signal as covariates. Linear trends were removed.

ALFF was computed based on the fast Fourier transform (FFT) and the time series of each voxel was transformed to the frequency domain without band-pass filtering. The square root was first calculated at each frequency of the power spectrum, and the mean square root was then obtained across frequency band of 0.01–0.08 Hz for each voxel. Finally, the ALFF of each voxel was divided by the global mean to acquire the mALFF.

### 2.5. Statistical analysis

Differences in the clinical and demographic characteristics between patients and controls were assessed using SPSS (v26.0, IBM, Chicago, NY, United States). The whole brain analysis of mALFF alteration in adolescent nsMDDs compared to HC was conducted through the SPM using the two-sample *t*-tests. Age, gender and education level was entered into the general linear model as covariates. An initial voxel-wise threshold was set at *p* < 0.05 with subsequent family-wise error (FWE) corrected value of *p* < 0.05 at the cluster level. Then, region of interest (ROI) analysis was conducted to compare the mean signal at the altered brain regions at pre and post-treatment.

The other whole brain analysis of mALFF changes in adolescent MDD at post-treatment compared to pre-treatment was conducted through the SPM using the paired *t*-test. An initial voxel-wise threshold was set at *p* < 0.05 with subsequent family-wise error (FWE) corrected value of *p* < 0.05 at the cluster level. Also, ROI analysis was conducted to compare the mean signal changes at the altered brain regions at pre and post-treatment.

Depression severity changes were further assessed through the paired *t*-test. Also, Pearson correlation was carried out to examine relationship between the mALFF alteration and depression severity changes. The xjView toolbox^3^ was used for imaging visualization.

## 3. Results

### 3.1. Participants and clinical characteristics

Fifteen adolescent nsMDDs (13 female/2 male, 14.60+/−1.35 years old) and twenty-two HC (13 female/9 male, 15.27 +/−1.05 years old) to be included into the analysis. The statistical analysis showed no significant differences of gender, age and education years between the two groups. More than half of adolescent nsMDDs had total HAMD scores higher than 24, corresponding to the severe status. The demographic and clinical characteristics of these individuals are shown in Table 1.

**Table 1:** Demographic and clinical characteristics of participants.

	nsMDDs (*n* = 15)	HC (*n* = 22)	Statistics
Gender, *n*			
Female	13	13	*p*^a^ = 0.141
Male	2	9	
Age, yrs	14.60 (1.35)	15.27 (1.05)	*t* = −1.20, *p* = 0.24
Education, yrs	9 (1.77)	9.36 (2.72)	*t* = −0.49, *p* = 0.63
HAMD^b^	23.53 (3.66)	1.59 (2.59)	*t* = 23.21, *p* < 0.001
*N*^c^, %	8 (53.3%)		
HAMD^d^	14.47 (6.29)	–	–
*N*^e^, %	1 (6.7%)		

HAMD, hamilton depression scale. ^a^indicates the Chi-square test, ^b^indicates the HAMD scores before treatment, ^d^indicates the HAMD scores after treatment, ^c,e^indicates the number of subjects with HAMD score > 24 before and after treatment, respectively.

### 3.2. Whole brain comparison of mALFF in adolescent nsMDDs

**Figure 1:**
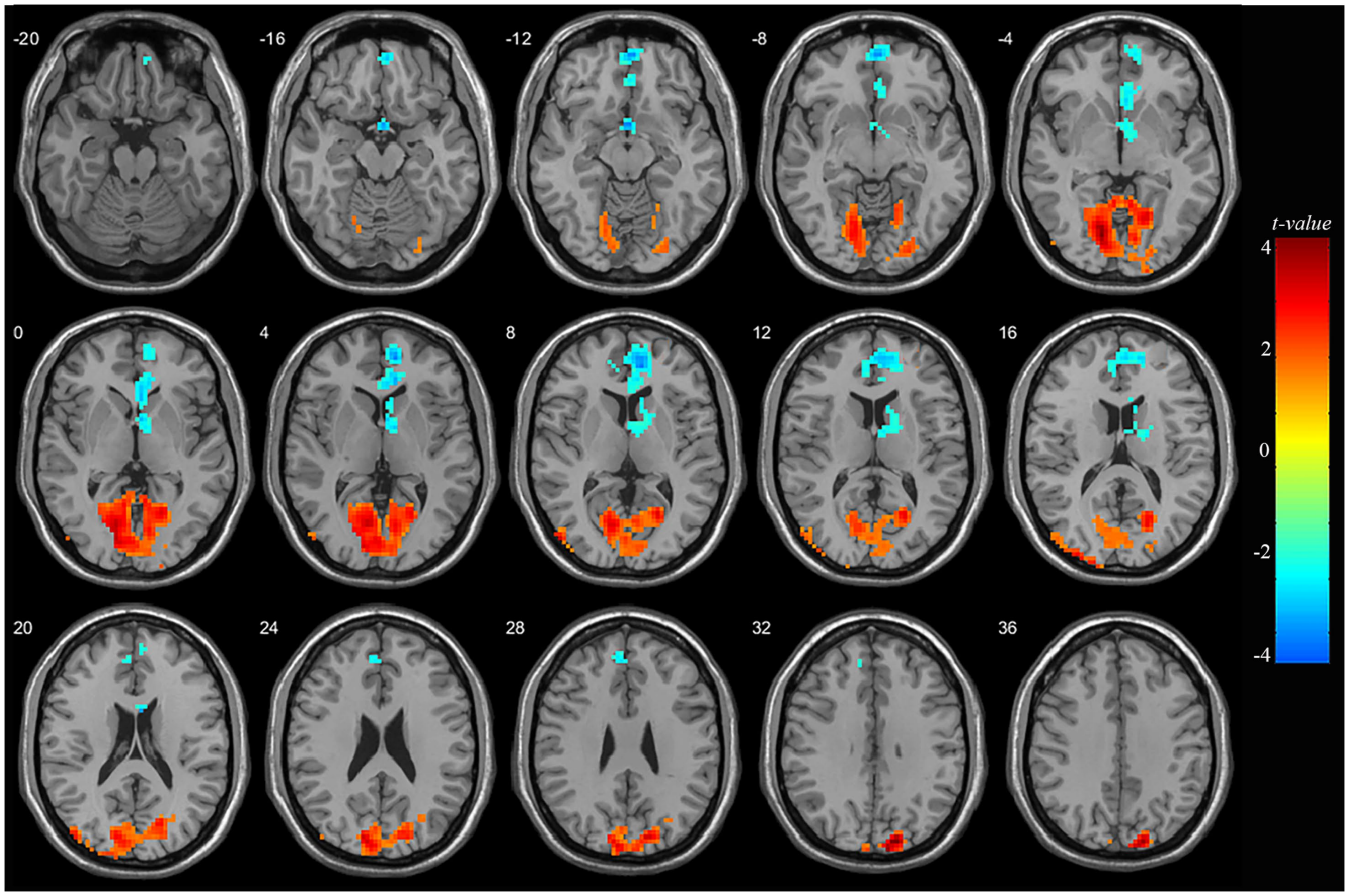
Adolescents with nsMDDs showed mALFF disturbances at frontal and occipital brain regions. Compared to controls, adolescent nsMDDs had significantly increased mALFF at right superior occipital extending to left lingual gyrus; Meanwhile decreased mALFF were found at right medial superior frontal extending to left medial orbital superior frontal. The color bar depicts the *t* value.

At pre-treatment, significant differences were observed in regional mALFF between adolescent nsMDDs and HC groups. Compared to controls, adolescent nsMDDs showed significantly increased mALFF in the right superior occipital extending to left lingual gyrus (Peak value = 4.60; at [15, −87, 33]; k = 1,565 voxels) at *p* < 0.05 (corrected) (see Figure 1; Table 2). The hyperactivity at occipital cortex still remained significantly at *p* < 0.001 (corrected) (see Supplementary Figure S1; Table S1). Meanwhile, the mALFF was found decreased at right medial superior frontal gryus (SFG, Peak value = −3.95; at [9, 51, 6]; k = 607 voxels) extending to left medial orbital SFG at *p* < 0.062 (corrected) (see Figure 1; Table 2).

**Table 2:** Locations of regional mALFF alteration in adolescent nsMDDs compared to controls.

Brain region	MNI coordinates of peak			Peak *t*-value	Spatial extent (in contiguous voxels)
	*x*	*y*	*z*		
*nsMDDs > HC*					
**R. Superior occipital**	15	−87	33	4.60	1,565
L. Lingual gyrus	−15	−75	−3	4.27	257
*nsMDDs < HC*					
**R. Medial superior frontal**	9	51	6	−3.95	607
L. Medial orbital superior frontal	0	57	−9	−3.29	37

### 3.3. Whole brain comparison of mALFF in adolescent nsMDDs before and after treatment

**Figure 2:**
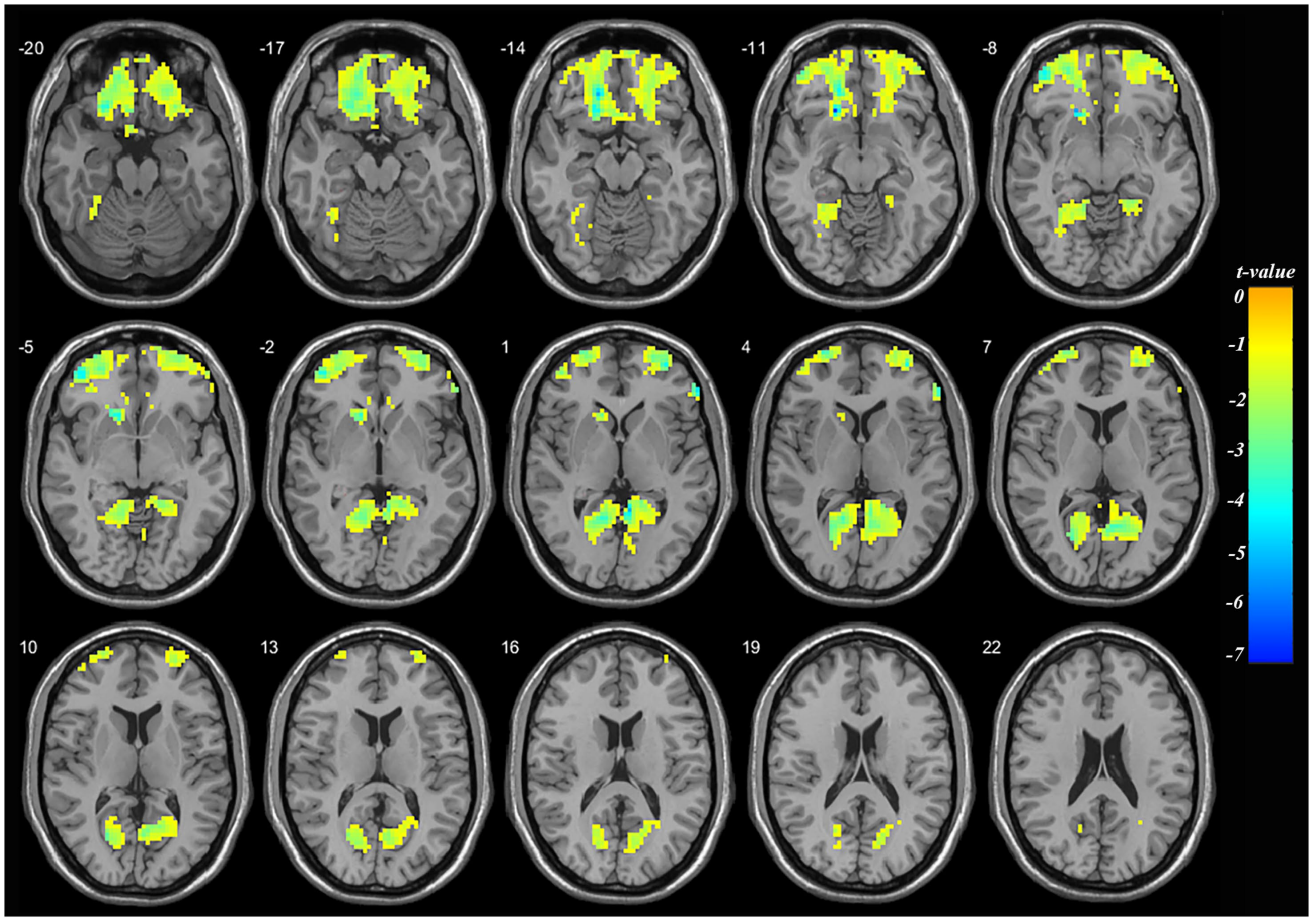
Adolescents with nsMDDs showed decreased mALFF at frontal and occipital brain regions after treatment. Compared to the pretreatment, adolescent nsMDDs had significantly decreased mALFF at left orbital middle frontal extending to right triangular inferior frontal, and bilateral lingual gyrus after treatment. The color bar depicts the *t* value.

Compared to pre-treatment, significantly decreased mALFF was found at left orbital middle frontal gyrus (orbMFG, Peak value = −7.34; at [−18, 36, −15]; k = 1,638 voxels) extending to right triangular inferior frontal gyrus (IFG) in adolescent nsMDDs after treatment at *p* < 0.05 (corrected). The reduced neuronal activity at the orbMFG remained significantly at *p* < 0.001 (see Supplementary Figure S1; Supplementary Table S1). Also, the significantly decreased mALFF was found at the left lingual gyrus (Peak value = −4.44; at [−15, −54, 0]; k = 814 voxels) extending to right lingual gyrus at *p* < 0.05 (corrected) (see Figure 2; Table 3).

**Table 3:** Locations of regional mALFF alteration in adolescent nsMDDs before and after treatment.

Brain region	MNI coordinates of peak			Peak *t*-value	Spatial extent (in contiguous voxels)
	*x*	*y*	*z*		
**L. Orbital middle frontal**	−18	36	−15	−7.34	1,638
R. Triangular inferior frontal	57	39	0	−5.41	15
**L. Lingual**	−15	−54	0	−4.44	814
R. Lingual	6	−60	6	−4.18	122

### 3.4. Comparison of mALFF at region of interests in adolescent nsMDDs before and after treatment

**Figure 3:**
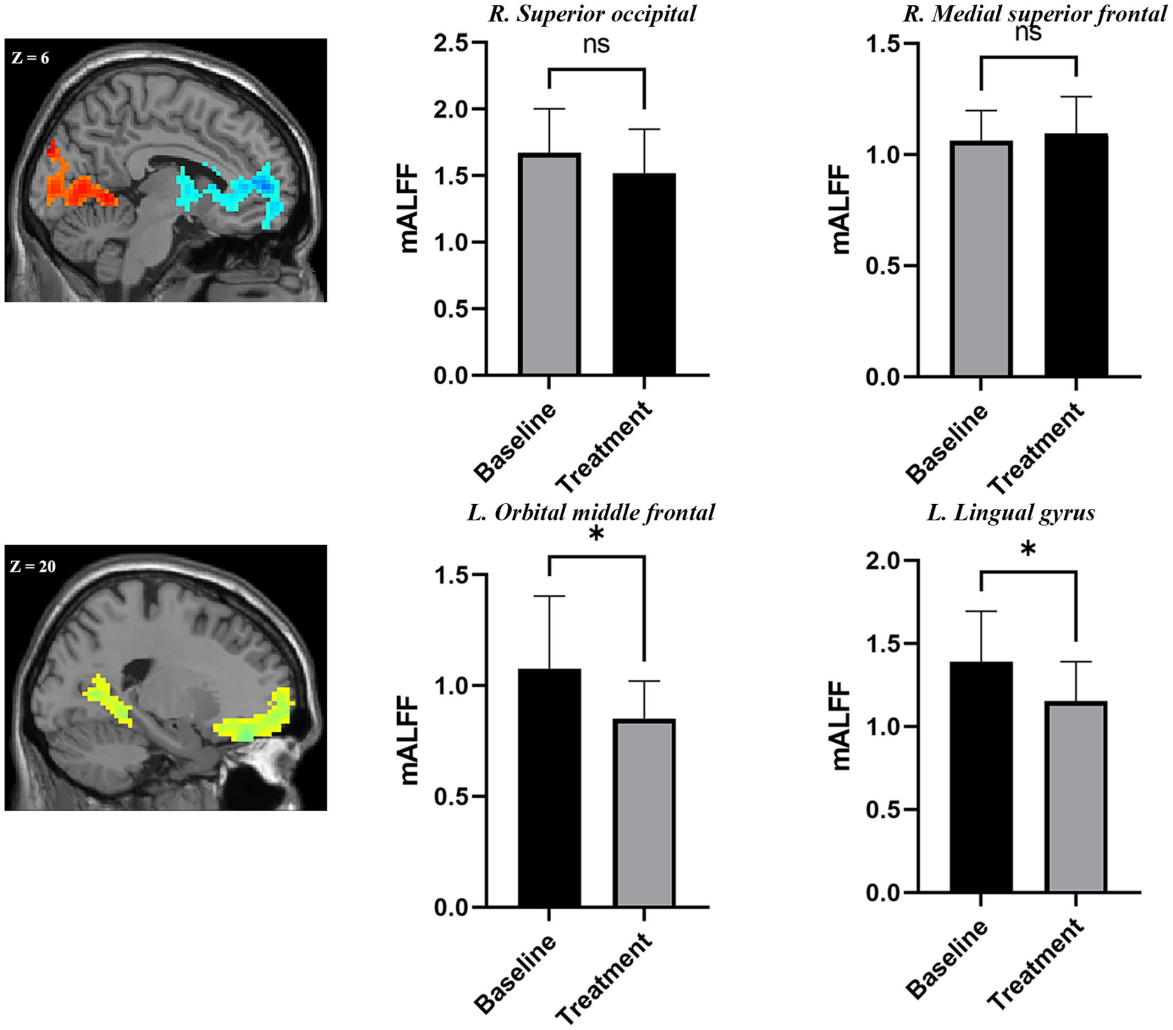
Adolescents with nsMDDs showed mean mALFF signal changes after treatment. The upper panel showed a trend of downregulation of mALFF at the occipital areas. At the mean time, a trend of upregulation of mALFF at the frontal areas. Further, compared to the pre-treatment, the adolescent nsMDDs showed significantly decreased mALFF at left orbital middle frontal and lingual gyrus after treatment. *p < 0.05.

The ROI analysis found a trend of decreased mALFF at the hyperactive occipital cortex found in adolescent nsMDDs after sertraline treatment. Whereas, a trend of increased mALFF at the hypoactive medial SFG found in adolescent nsMDDs after treatment. Further, ROI analysis found significantly decreased mALFF at left orbMFG in adolescent nsMDDs compared to pre-treatment after sertraline treatment. Also, the mALFF at lingual gyrus in adolescent nsMDDs showed significantly decreased compared to pre-treatment (see Figure 3).

### 3.5. Correlation with clinical severity changes in adolescent nsMDDs after treatment

After sertraline treatment, the HAMD scores were significantly decreased in adolescent nsMDDs (*t* = 5.86, *p* < 0.001) (see details in Supplementary Table S2). Further, Pearson correlation analysis showed no significant correlation between the HAMD changes with mALFF alteration in adolescent nsMDDs. But the correlation between the changes of mALFF at occipital cortex and HAMD was achieved nearly significant (r = −0.295, *p* = 0.287 for superior occipital; r = −0.357, *p* = 0.192 for lingual gyrus) (see Supplementary Table S3). Finally, the NSSI measured by OSI was 1.47 +/−0.64 before treatment, and after therapy, it showed a decrease (1.27 +/−0.96) but no significant difference was observed (t = 0.716, *p* = 0.486) (see Supplementary Table S4). Pearson correlation showed no significant correlation of the alteration in changes of mALFF and NSSI after treatment (see Supplementary Table S5).

## 4. Discussion

Through the rs-fMRI technique, the present study identified the abnormal neuronal activity pattern in adolescent nsMDDs. Further, we elaborated the changes of this activity pattern after the eight-week sertraline therapy. Compared with healthy individuals, adolescent nsMDDs had increased neuronal spontaneous activity at superior occipital cortex, and decreased activity at medial SFG. Further, no significant differences but a trend of decreased activity were observed at the superior occipital cortex in adolescent nsMDDs after treatment. At the same time, a trend of increased activity was observed at the medial SFG after treatment. The changes of neuronal activity at fronto-occipital areas reflect the downregulation and upregulation of antidepressant effects. Moreover, the neuronal activity at left orbMFG and lingual gyrus was significantly decreased after treatment, which might indicate the decreased NSSI behaviors or thoughts, and anxiety-depression level in adolescent nsMDDs. Finally, significantly decreased HAMD scores were found in adolescent nsMDDs after the sertraline therapy.

### 4.1. Findings of mALFF alteration in adolescent nsMDDs

The occipital cortex is involved in primary visual stimulation and is associated with facial emotional awareness and working memory (22). Previous studies have provided evidences of the decreased concentration of gamma-aminobutyric acid (GABA) at the occipital cortex (23–25). As GABA is an inhibitory neurotransmitter, the deficits might cause the abnormal spontaneous activity found in nsMDDs.

Located at the occipital cortex, lingual gyrus plays an important role in emotional processing, logical reasoning, and visual information integration (26, 27). Previous studies proposed the activation in the lingual gyrus in MDD patients may be a sign of dysfunction in emotion processing and involved in suicidal behavior (28, 29). Furthermore, Yan et al. (30) demonstrated that nsMDDs have increased ALFF in the lingual gyrus and they speculated that increased resting-state activity in the lingual gyrus was associated with NSSI behavior. In this study, we found that nsMDDs exhibited increased mALFF in the right superior occipital gyrus and left lingual gyrus compared with HC, which was consistent with previous studies. Besides, the left lingual gyrus is involved in linguistic processing (31, 32) and an additional study indicated that the changes in speech characteristics could predict depression severity (33). These findings could explain the language expression problems such as reduced speech rate and decreased volume of tone in MDD patients.

Moreover, we found that nsMDDs exhibited decreased mALFF in the medial SFG. The SFG is the core region of the default mode network (DMN), involved in memory-related cognitive and motor control tasks, as well as self-related processing (34). Previous studies have reported abnormalities in DMN of MDD patients, and this abnormality could be related with suicidal ideation and NSSI behavior (15, 35). The decreased levels of glutamatergic (Glu) metabolites in the medial frontal cortex were linked with the pathophysiology of depression (36). Contrary to GABA, Glu is an excitatory neurotransmitter, and the deficits might cause the decreased neuronal activity found in the study.

### 4.2. Findings of mALFF changes in adolescent nsMDDs after sertraline treatment

The left orbMFG is located in the prefrontal lobe, and belongs to the reward network involved with decision making processes. Sun et al. (37) found that the decreased activation in this region was associated with the efficacy of antidepressant treatment, which was similar to our results. NSSI behavior is associated with decision making, the decreased activation found in this region might indicate the reduction of NSSI behaviors or thoughts. It is inferred that the significantly reduced HAMD score after treatment might be associated with the reduced NSSI behaviors or thoughts.

The IFG plays an important role in emotional and cognitive control. As an important component of the IFG, the right triangular part is suggested taking part in language comprehension (38). Also, this region was involved in unconscious information processing (39). Further, it was also associated with the early-onset depression (EOD) and late -onset depression (LOD). Zhang et al. (40) found the EOD patients exhibited increased activation in the right triangular IFG compared with the LOD patients. They indicated that the specific brain alterations of EOD and LOD patients could be associated with different clinical symptoms. EOD patients are associated with more suicidal behaviors and thoughts, slower treatment response and higher prevalence of co-morbid personality disorders (40–42). According to these reports, we speculated that the decreased activation in the right triangular IFG was linked with the reduced suicidal behaviors and the efficacy of the sertraline therapy.

Also, nsMDDs exhibited decreased mALFF at the bilateral lingual gyrus after sertraline therapy, which could indicate that the abnormal activation in the occipital gyrus of the patient was relieved after treatment. It has been demonstrated that SSRI treatments could increase the GABA concentration at the occipital cortex in depressed patients (43, 44). With the increased GABA concentration, it could further lead to the decreased functional neuronal activity in the occipital cortex. Therefore, this GABA concentration improvement could be a potential mechanism underlying the sertraline therapy in nsMDDs to diminish the hyperactivity at the occipital cortex.

Moreover, the ROI analysis found downregulation of hyperactivity at the superior occipital cortex and upregulation of hypoactivity at the medial SFG in adolescent nsMDDs after treatment. It is widely acknowledged that the breakdown in brain serotonin (5-HT) signaling is closely related to depression symptoms, and antidepressants have been developed according to this basis (45, 46). Sertraline is an SSRI, which plays an important role in treating depression through regulating the abnormal 5-HT. Therefore, the changes of neuronal activity might indicate the efficacy of sertraline therapy.

Over all, our findings of neuronal activity alteration pattern in nsMDDs were consistent with the previous studies. Also, there were few studies that used the rs-fMRI technique to examine the efficacy of sertraline treatment in adolescent nsMDDs. But there were still some limitations in this study. First, our sample size was relatively small and some participants could not endure long-term MRI examinations. The future study with large sample size could be carried out to replicate and validate our findings. Second, nsMDDs exhibited changes of neuronal activity as well as HAMD scores after the therapy, but there was no significant correlation between these changes. While a previous study based on structural imaging has indicated that the alterations in the first week of treatment might be associated with long-term treatment efficacy (47). Thus, a longitudinal observation would be conducted in the future study. Lastly, some participants did not fully understand the OSI report, thus, we could not exactly determine the frequency of NSSI. We would optimize the procedure in the future study.

## 5. Conclusion

In summary, this study revealed that sertraline was an effective way to treat nsMDDs in adolescents through the direct insight into sertraline-induced brain spontaneous activity changes using the rs-fMRI technique. Specifically, it exhibited downregulation of the hyperactivity at superior occipital cortex and upregulation of the hypoactivity at medial SFG in adolescent nsMDDs. Alongside the regulation effects, significantly decreased neuronal functional activity in the decision related orbMFG, and anxiety-depression related lingual gyrus implied the reduced NSSI behaviors or thoughts in adolescent nsMDDs.

## Data availability statement

The raw data supporting the conclusions of this article will be made available by the authors, without undue reservation.

## Ethics statement

This study was approved by the Human Research and Ethics Committee of the First Affiliated Hospital of Chongqing Medical University (no. 2021-546). Written informed consent to participate in this study was provided by the participants’ legal guardian/next of kin.

## Author contributions

LD: conceptualization, formal analysis, and writing – original draft. XZ: formal analysis, visualization, and writing – review and editing. RY and XW: imaging scanning. FD and XL: data collection. LK: project administration, funding acquisition, and writing – review and editing. All authors contributed to the article and approved the submitted version.

## Funding

This work received the funding from National Natural Science of Foundation of China (NSFC – 81971286 and 81671360). This work was also supported by the Chongqing Medical University Postdoctoral Bridging Fellowship.

## Conflict of interest

The authors declare that the research was conducted in the absence of any commercial or financial relationships that could be construed as a potential conflict of interest.

## Publisher’s note

All claims expressed in this article are solely those of the authors and do not necessarily represent those of their affiliated organizations, or those of the publisher, the editors and the reviewers. Any product that may be evaluated in this article, or claim that may be made by its manufacturer, is not guaranteed or endorsed by the publisher.

## Footnotes

^1^ http://www.fil.ion.ucl.ac.uk/spm

^2^ http://www.restfmri.net/forum/REST

^3^ https://www.alivelearn.net/xjview

## References

[1] American Psychiatric Association. Diagnostic and statistical manual of mental disorders. 5th ed. Washington DC: American Psychiatric Association Press (2013).

[2] Costello EJ Copeland W Angold A. Trends in psychopathology across the adolescent years: what changes when children become adolescents, and when adolescents become adults? J Child Psychol Psychiatry. (2011) 52:1015–25. doi: 10.1111/j.1469-7610.2011.02446.x, PMID: 21815892 PMC3204367

[3] Johnson D Dupuis G Piche J Clayborne Z Colman I. Adult mental health outcomes of adolescent depression: a systematic review. Depress Anxiety. (2018) 35:700–16. doi: 10.1002/da.22777 29878410

[4] Garisch JA Wilson MS. Prevalence, correlates, and prospective predictors of non-suicidal self-injury among New Zealand adolescents: cross-sectional and longitudinal survey data. Child Adolesc Psychiatry Ment Health. (2015) 9:28. doi: 10.1186/s13034-015-0055-6, PMID: 26157484 PMC4495816

[5] Plener PL Allroggen M Kapusta ND Brähler E Fegert JM Groschwitz RC. The prevalence of nonsuicidal self-injury (NSSI) in a representative sample of the German population. BMC Psychiatry. (2016) 16:353. doi: 10.1186/s12888-016-1060-x, PMID: 27760537 PMC5069807

[6] Ghinea D Edinger A Parzer P Koenig J Resch F Kaess M. Non-suicidal self-injury disorder as a stand-alone diagnosis in a consecutive help-seeking sample of adolescents. J Affect Disord. (2020) 274:1122–5. doi: 10.1016/j.jad.2020.06.009, PMID: 32663940

[7] Zang YF He Y Zhu CZ Cao QJ Sui MQ Liang M. Altered baseline brain activity in children with ADHD revealed by resting-state functional MRI. Brain and Development. (2007) 29:83–91. doi: 10.1016/j.braindev.2006.07.002, PMID: 16919409

[8] Fang J-W Yu Y-J Tang L-Y Chen S-Y Zhang M-Y Sun T. Abnormal fractional amplitude of low-frequency fluctuation changes in patients with monocular blindness: a functional magnetic resonance imaging (MRI) study. Med Sci Monit. (2020) 26:e926224. doi: 10.12659/msm.926224, PMID: 32773731 PMC7439597

[9] Gong J Wang J Qiu S Chen P Luo Z Wang J. Common and distinct patterns of intrinsic brain activity alterations in major depression and bipolar disorder: voxel-based meta-analysis. Transl Psychiatry. (2020) 10:353–13. doi: 10.1038/s41398-020-01036-5, PMID: 33077728 PMC7573621

[10] Lee J Pavuluri MN Kim JH Suh S Kim I Lee MS. Resting-state functional connectivity in medication-naive adolescents with major depressive disorder. Psychiatry Res Neuroimaging. (2019) 288:37–43. doi: 10.1016/j.pscychresns.2019.04.008, PMID: 31071543

[11] Sacchet MD Ho TC Connolly CG Tymofiyeva O Lewinn K.Z Han LK. Large-scale hypoconnectivity between resting-state functional networks in unmedicated adolescent major depressive disorder. Neuropsychopharmacology, (2016) 41:2951–2960. doi: 10.1038/npp.2016.76, PMID: 27238621 PMC5061890

[12] Wang X Zhou H Zhu X. Attention deficits in adults with Major depressive disorder: A systematic review and meta-analysis Asian J Psychiatr, (2020) [online] 53:102359. doi: 10.1016/j.ajp.2020.102359, PMID: 32891927

[13] Plener PL Bubalo N Fladung AK Ludolph AG Lulé D. Prone to excitement: adolescent females with non-suicidal self-injury (NSSI) show altered cortical pattern to emotional and NSS-related material. Psychiatry Res Neuroimaging. (2012) 203:146–52. doi: 10.1016/j.pscychresns.2011.12.012, PMID: 22901627

[14] Huang Q Xiao M Ai M Chen J Wang W Hu L. Disruption of neural activity and functional connectivity in adolescents with major depressive disorder who engage in non-suicidal self-injury: a resting-state fMRI study. Front Psych. (2021) 12:571532. doi: 10.3389/fpsyt.2021.571532, PMID: 34140897 PMC8203805

[15] Zhou Y Yu R Ai M Cao J Li X Hong S. A resting state functional magnetic resonance imaging study of unmedicated adolescents with non-suicidal self-injury behaviors: evidence from the amplitude of low-frequency fluctuation and regional homogeneity indicator. Front Psych. (2022) 13:925672. doi: 10.3389/fpsyt.2022.925672, PMID: 35782416 PMC9247173

[16] Turner JB Austin BS Chapma LA. Treating nonsuicidal self-injury: a systematic review of psychological and pharmacological interventions. Can J Psychiatry. (2014) 59:576–85. doi: 10.1177/070674371405901103, PMID: 25565473 PMC4244876

[17] Santamarina-Perez P Romero S Mendez I Leslie SM Packer MM Sugranyes G. Fronto-limbic connectivity as a predictor of improvement in nonsuicidal self-injury in adolescents following psychotherapy. J Child Adolesc Psychopharmacol. (2019) 29:456–65. doi: 10.1089/cap.2018.0152, PMID: 31225733

[18] Cullen KR Schreiner MW Klimes-Dougan B Eberly LE LaRiviere LL Lim KO. Neural correlates of clinical improvement in response to N-acetylcysteine in adolescents with non-suicidal self-injury. Prog Neuro-Psychopharmacol Biol Psychiatry. (2020) 99:109778. doi: 10.1016/j.pnpbp.2019.109778, PMID: 31682891 PMC7058485

[19] Furukawa TA Cipriani A Cowen PJ Leucht S Egger M Salanti G. Optimal dose of selective serotonin reuptake inhibitors, venlafaxine, and mirtazapine in major depression: a systematic review and dose-response meta-analysis. Lancet Psychiatry. (2019) 6:601–9. doi: 10.1016/s2215-0366(19)30217-2, PMID: 31178367 PMC6586944

[20] Sharp SC Hellings JA. Efficacy and safety of selective serotonin reuptake inhibitors in the treatment of depression in children and adolescents. Clin Drug Investig. (2006) 26:247–55. doi: 10.2165/00044011-200626050-00002 17163258

[21] Wagner KD. Efficacy of sertraline in the treatment of children and adolescents with major depressive disorder two randomized controlled trials. JAMA. (2003) 290:1033–41. doi: 10.1001/jama.290.8.1033, PMID: 12941675

[22] Shan X Cui X Liu F Li H Huang R Tang Y. Shared and distinct homotopic connectivity changes in melancholic and non-melancholic depression. J Affect Disord. (2021) 287:268–75. doi: 10.1016/j.jad.2021.03.038, PMID: 33799047

[23] Bhagwagar Z. Wylezinska M. Jezzard P. Evans J. Boorman E., M. Matthews P.. (2007). Low GABA concentrations in occipital cortex and anterior cingulate cortex in medication-free, recovered depressed patients. Int J Neuropsychopharmacol, 11, 255–260. doi: 10.1017/s1461145707007924, PMID: 17625025

[24] Song Z Huang P Qiu L Wu Q Gong Q Zhang B. Decreased occipital GABA concentrations in patients with first-episode major depressive disorder:a magnetic resonance spectroscopy study. J Biomed Eng. (2012) 29:233–6. PMID: 22616164

[25] Song XM Hu X-W Li Z Gao Y Ju X Liu D-Y. Reduction of higher-order occipital GABA and impaired visual perception in acute major depressive disorder. Mol Psychiatry. (2021) 26:6747–55. doi: 10.1038/s41380-021-01090-5, PMID: 33863994 PMC8760062

[26] Geng J Yan R Shi J Chen Y Mo Z Shao J. Altered regional homogeneity in patients with somatic depression: a resting-state fMRI study. J Affect Disord. (2019) 246:498–505. doi: 10.1016/j.jad.2018.12.066, PMID: 30599374

[27] Zhou J Yao N Fairchild G Zhang Y Wang X. Altered hemodynamic activity in conduct disorder: a resting-state fMRI investigation. PLoS One. (2015) 10:e0122750. doi: 10.1371/journal.pone.0122750, PMID: 25816069 PMC4376798

[28] Simeonova D Paunova R Stoyanova K Todeva-Radneva A Kandilarova S Stoyanov D. Functional MRI correlates of Stroop N-Back test underpin the diagnosis of major depression. J Integr Neurosci. (2022) 21:113. doi: 10.31083/j.jin2104113, PMID: 35864765

[29] Zhang S Chen J Kuang L Cao J Zhang H Ai M. Association between abnormal default mode network activity and suicidality in depressed adolescents. BMC Psychiatry. (2016) 16:337. doi: 10.1186/s12888-016-1047-7, PMID: 27688124 PMC5041526

[30] Yan R Huang Y Shi J Zou H Wang X Xia Y. Alterations of regional spontaneous neuronal activity and corresponding brain circuits related to non-suicidal self-injury in young adults with major depressive disorder. J Affect Disord. (2022) 305:8–18. doi: 10.1016/j.jad.2022.02.040, PMID: 35181386

[31] Palejwala AH Dadario NB Young IM O’Connor K Briggs RG Conner AK. Anatomy and white matter connections of the lingual gyrus and cuneus. World Neurosurg. (2021) 151:e426–37. doi: 10.1016/j.wneu.2021.04.050, PMID: 33894399

[32] Zhang C Lee TMC Fu Y Ren C Chan CCH Tao Q. Properties of cross-modal occipital responses in early blindness: an ALE meta-analysis. Neuroimage Clin. (2019) 24:102041. doi: 10.1016/j.nicl.2019.102041, PMID: 31677587 PMC6838549

[33] Homan S Gabi M Klee N Bachmann S Moser A-M Duri M. Linguistic features of suicidal thoughts and behaviors: a systematic review. Clin Psychol Rev. (2022) 95:102161. doi: 10.1016/j.cpr.2022.102161, PMID: 35636131

[34] Si L Cui B Li Z Li X Li K Ling X. Concurrent brain structural and functional alterations in patients with chronic unilateral vestibulopathy. Quant Imaging Med Surg. (2022) 12:3115–25. doi: 10.21037/qims-21-655, PMID: 35655817 PMC9131349

[35] Ho TC Walker JC Teresi GI Kulla A Kirshenbaum JS Gifuni AJ. Default mode and salience network alterations in suicidal and non-suicidal self-injurious thoughts and behaviors in adolescents with depression. Transl Psychiatry. (2021) 11:38. doi: 10.1038/s41398-020-01103-x, PMID: 33436537 PMC7804956

[36] Moriguchi S Takamiya A Noda Y Horita N Wada M Tsugawa S. Glutamatergic neurometabolite levels in major depressive disorder: a systematic review and meta-analysis of proton magnetic resonance spectroscopy studies. Mol Psychiatry. (2019) 24:952–64. doi: 10.1038/s41380-018-0252-9, PMID: 30315224 PMC6755980

[37] Sun J Ma Y Du Z Wang Z Guo C Luo Y. Immediate modulation of transcutaneous auricular Vagus nerve stimulation in patients with treatment-resistant depression: a resting-state functional magnetic resonance imaging study. Front Psych. (2022) 13:923783. doi: 10.3389/fpsyt.2022.923783, PMID: 35845466 PMC9284008

[38] Yang H Zhang J Cheng J. Effects of donepezil on the amplitude of low-frequency fluctuations in the brain of patients with Alzheimer’s disease: evidence from resting-state functional magnetic resonance imaging. Neuroreport. (2021) 32:907–12. doi: 10.1097/wnr.0000000000001659, PMID: 34029287 PMC8253505

[39] Shi J Huang H Jiang R Mao X Huang Q Li A. The right inferior frontal gyrus plays an important role in unconscious information processing: activation likelihood estimation analysis based on functional magnetic resonance imaging. Front Neurosci. (2022) 16:781099. doi: 10.3389/fnins.2022.781099, PMID: 35401077 PMC8987111

[40] Zhang Z Chen Y Wei W Yang X Meng Y Yu H. Changes in regional homogeneity of medication-free major depressive disorder patients with different onset ages. Front Psych. (2021) 12:713614. doi: 10.3389/fpsyt.2021.713614, PMID: 34658953 PMC8517084

[41] Bukh JD. Differences between early and late onset adult depression. Clin Pract Epidemiol Ment Health. (2011) 7:140–7. doi: 10.2174/1745017901107010140, PMID: 21866230 PMC3158434

[42] Herzog DP Wagner S Engelmann J Treccani G Dreimüller N Müller MB. Early onset of depression and treatment outcome in patients with major depressive disorder. J Psychiatr Res. (2021) 139:150–8. doi: 10.1016/j.jpsychires.2021.05.048, PMID: 34058654

[43] Sanacora G Fenton LR Fasula MK Rothman DL Levin Y Krystal JH. Cortical *γ*-aminobutyric acid concentrations in depressed patients receiving cognitive behavioral therapy. Biol Psychiatry. (2006) 59:284–6. doi: 10.1016/j.biopsych.2005.07.015, PMID: 16139814

[44] Sanacora G Mason GF Rothman DL Krystal JH. Increased occipital cortex GABA concentrations in depressed patients after therapy with selective serotonin reuptake inhibitors. Am J Psychiatr. (2002) 159:663–5. doi: 10.1176/appi.ajp.159.4.663, PMID: 11925309

[45] Sharp T Cowen PJ. 5-HT and depression: is the glass half-full? Curr Opin Pharmacol. (2011) 11:45–51. doi: 10.1016/j.coph.2011.02.003, PMID: 21377932

[46] Tan T Xu Z Gao C Shen T Li L Chen Z. Influence and interaction of resting state functional magnetic resonance and tryptophan hydroxylase-2 methylation on short-term antidepressant drug response. BMC Psychiatry. (2022) 22:218. doi: 10.1186/s12888-022-03860-z, PMID: 35337298 PMC8957120

[47] Bartlett EA DeLorenzo C Sharma P Yang J Zhang M Petkova E. Pretreatment and early-treatment cortical thickness is associated with SSRI treatment response in major depressive disorder. Neuropsychopharmacology. (2018) 43:2221–30. doi: 10.1038/s41386-018-0122-9, PMID: 29955151 PMC6135779

