# Supplementary material for: Abnormal brain spontaneous activity in major depressive disorder adolescents with non-suicidal self injury and its changes after sertraline therapy
Source: Front Psychiatry. 2023 Jun 13;14:1177227. doi: 10.3389/fpsyt.2023.1177227 (PMC10293671; doi:10.3389/fpsyt.2023.1177227)
Supplement: Supplementary file 1 [file Data_Sheet_1.docx]

Supplementary Material

# Supplementary Figures and Tables

## Whole brain comparison of mALFF in adolescent nsMDDs before and after treatment

At pre-treatment, significantly increased mALFF was found at left lingual gyrus extending to calcarine of occipital cortex (Peak value = 4.27; at [-15, -75, -3]; k = 51 voxels) in adolescent nsMDDs compared to HC at p < 0.001 (corrected). After sertraline treatment, significantly decreased mALFF was found at left orbital middle frontal (Peak value = -6.01; at [-18, 36, -15]; k = 44 voxels) in adolescent nsMDDs compared to pre-treatment at p < 0.001 (corrected) (see ***Supplementary Figure 1 & Table1***).


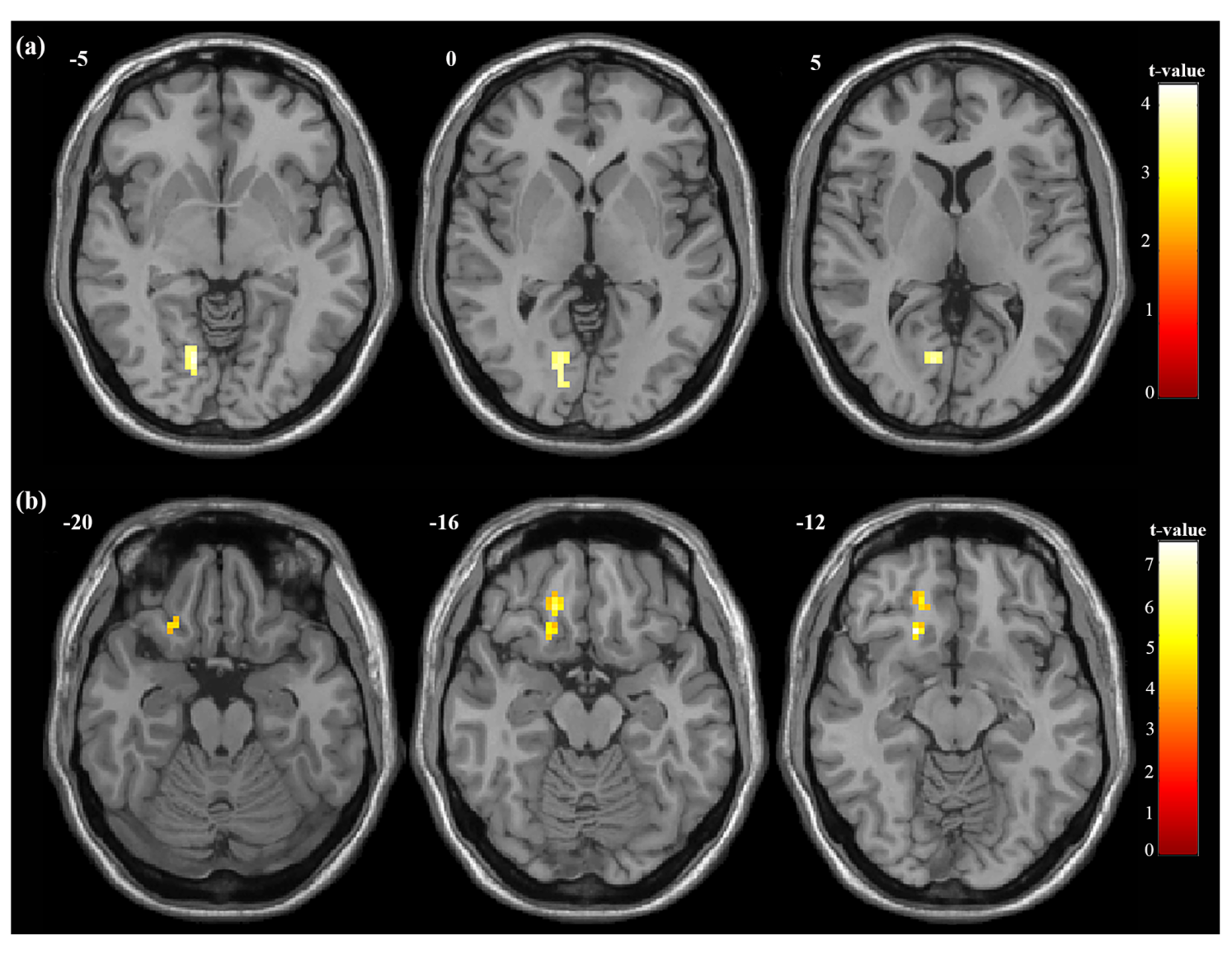


**Supplementary Figure 1.** The mALFF alteration in adolescent nsMDDs at pre and post-treatment. (a) Increased mALFF was found at left lingual extending to left calcarine in nsMDDs group before treatment. (b) Decreased mALFF was found at left orbital middle frontal in nsMDDs group after treatment. The color bar depicts the t-value.

**Supplementary Table 1**. Locations of regional mALFF alteration in adolescent nsMDDs.

| Brain region | MNI coordinates of peak | | | **Peak t-value** | **Spatial extent**  **(in contiguous voxels)** |
| --- | --- | --- | --- | --- | --- |
|  | x | y | z |  |  |
| **Increased mALFF at pre-treatment** | | | | | |
| **L. Lingual** | -15 | -75 | -3 | 4.27 | 51 |
| L. Calcarine | -12 | -72 | 6 | 3.82 | 9 |
| **Decreased mALFF at post-treatment** | | | | | |
| L. Orbital middle frontal | -18 | 36 | -15 | -6.01 | 44 |

## Depression severity changes and correlation with mALFF alteration in adolescent nsMDDs

The depression severity was significantly decreased in adolescent nsMDDs after treatment (see ***Supplementary Table 2***).

**Supplementary Table 2**. Changes of clinical severity in adolescent nsMDDs after treatment.

|  | Pre-treatment | Post-treatment | T(orx²) | P-value |
| --- | --- | --- | --- | --- |
| HAMD | 23.53 (3.66) | 14.47 (6.29) | 5.859 | ＜ 0.001 |

Pearson correlation was further conducted to examine the relationship between the changes of depression severity and mALFF in adolescent nsMDDs (see ***Supplementary Table 3***).

**Supplementary Table 3**. Pearson correlation between changes of mALFF and clinical severity.

| Brain region | mALFF_pre_ | mALFF_post_ | ∆mALFF/mALFF_pre_ | ∆HAMD/HAMD_pre_ | |
| --- | --- | --- | --- | --- | --- |
|  |  |  |  | Pearson r | P-value |
| **R. Superior occipital** | 1.674(0.328) | 1.52(0.33) | -0.065 (0.246) | -0.295 | 0.287 |
| **R. Medial superior frontal** | 1.062(0.136) | 1.094(0.167) | 0.036 (0.132) | 0.121 | 0.667 |
| **L. Orbital middle frontal** | 1.077(0.328) | 0.851(0.169) | -0.187(0.109) | -0.165 | 0.556 |
| **L. Lingual** | 1.389(0.306) | 1.153(0.238) | -0.155(0.147) | -0.357 | 0.192 |

*Note: mALFF_pre_ denotes the mALFF at pre-treatment; mALFF_post_ denotes the mALFF at post-treatment.

HAMD_pre_ denotes the HAMD at pre-treatment; ∆ denotes the changes.

## NSSI changes and correlation with mALFF alteration in adolescent nsMDDs

The NSSI measured by Ottawa self-injury inventory (OSI) was decreased in adolescent nsMDDs after treatment, but it showed no significant differences (see ***Supplementary Table 4***).

**Supplementary Table 4**. Changes of NSSI in adolescent nsMDDs after treatment.

|  | Pre-treatment | Post-treatment | T(orx²) | P-value |
| --- | --- | --- | --- | --- |
| NSSI | 1.47 (0.64) | 1.27 (0.96) | 0.716 | 0.486 |

Pearson correlation was conducted to examine the relationship between the changes of NSSI and mALFF in adolescent nsMDDs (see ***Supplementary Table 5***).

**Supplementary Table 5**. Pearson correlation between changes of mALFF and NSSI.

| Brain region | mALFF_pre_ | mALFF_post_ | ∆mALFF/mALFF_pre_ | ∆NSSI/NSSI_pre_ | |
| --- | --- | --- | --- | --- | --- |
|  |  |  |  | Pearson r | P-value |
| **R. Superior occipital** | 1.674(0.328) | 1.52(0.33) | -0.065 (0.246) | -0.003 | 0.991 |
| **R. Medial superior frontal** | 1.062(0.136) | 1.094(0.167) | 0.036 (0.132) | -0.015 | 0.958 |
| **L. Orbital middle frontal** | 1.077(0.328) | 0.851(0.169) | -0.187(0.109) | 0.155 | 0.583 |
| **L. Lingual** | 1.389(0.306) | 1.153(0.238) | -0.155(0.147) | -0.129 | 0.646 |

*Note:NSSI_pre_ denotes the NSSI at pre-treatment; ∆ denotes the changes.
